# School‐based education to prevent bullying in high schools in Indonesia

**DOI:** 10.1111/ped.14475

**Published:** 2021-03-22

**Authors:** Tamaki Noboru, Emmy Amalia, Paul Michael R Hernandez, Lina Nurbaiti, Wahyu Sulistya Affarah, Daisuke Nonaka, Rie Takeuchi, Hamsu Kadriyan, Jun Kobayashi

**Affiliations:** ^1^ Department of Global Health Graduate School of Health Sciences University of the Ryukyus Okinawa Japan; ^2^ Faculty of Medicine Mataram University Mataram Indonesia; ^3^ College of Public Health University of the Philippines Manila Manila Philippines; ^4^ Japanese Consortium for Global school Health and Research Okinawa Japan

**Keywords:** bullying, Indonesia, Islamic education, religious education, school health

## Abstract

**Background:**

The Global School‐based Health Survey showed that 20.6% of Indonesian students aged 13–17 years old were bullied. The proportion was lower than those reported from Southeast Asian countries (28.3–51.0%). School education was reported to contribute to the reduction of bullying, but no similar study has been done in Indonesia. This study aimed to explore the role of school‐based education in preventing bullying in high schools in Indonesia.

**Methods:**

In‐depth interviews with principals and focus group discussions with teachers from five schools in Mataram City were conducted in 2018. Data were transcribed and analyzed using thematic analysis. Participant observations and document reviews were conducted to verify the data.

**Results:**

Seven themes emerged: (i) Bullying as a problem; (ii) Causes of bullying; (iii) Effects of bullying; (iv) Curricular interventions; (v) Cultural interventions; (vi) Institutional interventions; and (vii) Challenges and recommendations regarding current interventions. Curricular interventions include *Pancasila* (state ideology or principles of the state’s philosophy) and civic education, and religious education, while cultural interventions include cultural practices and extracurricular activities. The schools provide an enabling environment by maintaining a good physical environment and implementing policies to prevent bullying. These activities promote the prevention of school bullying.

**Conclusions:**

This study demonstrated that moral education in curriculum and cultural activities are avenues for the prevention of school bullying. The implementation of both religious education and civic education encouraged the creation of values among students. In Indonesia, current interventions should be continued and must be modified to respond with societal changes.

Youth violence is a global public health problem. It includes a range of acts from bullying and physical fighting to more severe sexual and physical assault, and homicide.^1^ Data show that 32% of students were bullied in some form by their peers at school on one or more days over the period of a month.^2^ In particular, school bullying is a widespread phenomenon in many countries^3^, ^4^ and has been linked to increased suicide rates among adolescents.^5^, ^6^ Bullying can result in physical injury, social and emotional distress, and even death.^7^, ^8^ Victimized youth are at increased risk of depression, anxiety, sleep difficulties, insecurity, loneliness, unhappiness, physical and mental symptoms, low self‐esteem, and poor school adjustment,^9^, ^10^ and those who bully others are at increased risk for substance use, academic problems, conduct problems, and violence later in life.^11^, ^12^ Victims of bullying suffer the most serious consequences and are at greater risk for mental health and behavior problems.^13^

Systematic reviews concluded that bullying prevention programs in schools work.^14^, ^15^ A previous study indicated that school‐based bullying prevention programs such as parent meetings, firm disciplinary methods, improved playground supervision, and whole‐school programs were effective against bullying.^16^ The literature suggests that preventive intervention should be reinforced by the inclusion of whole community awareness campaigns about the nature of bullying and its dangers.^17^ The World Health Organization also recommends school‐based academic and social skills development to prevent bullying,^18^ and moral development to prevent violence during adolescence and early adulthood.^19^ Moral education is integrated into the curriculum in many countries, specifically in values education, citizenship education, and religious education.^20^ It often includes the prevention of bullying.^21^

Previous studies have revealed that Islamic countries, including Indonesia, tend to have lower rates of suicide compared with other countries.^22^, ^23^ The 2015 Global School‐based Health Survey showed that the percentage of Indonesian students aged 13–17 years old who were bullied was only 20.6%, whereas the prevalence of bullying among students of the same age group in Southeast Asian countries ranged from 28.3 to 51.0%.^24^ The difference in the prevalence of bullying might be explained by two specific characteristics of Indonesia. First, Indonesia has the world's largest Muslim population. Muslim teaching includes, for example, that “There shall be no infliction of harm on oneself or others.”^25^ Second, Indonesia is highly ethnically diverse, with more than 300 ethnic groups. Students are taught to respect others despite various differences. According to literature, school bullying can be caused by differences in race, sexuality, religion, disabilities and abilities, weight, height, or anything that creates a difference between one child and another.^26^ School‐based education in Indonesia is therefore considered to play a critical role in preventing bullying at school. However, such roles remain poorly understood.^27^ This study aimed to explore the role of school‐based education in preventing bullying in public high schools in Indonesia.

## Methods

### Study design

This study utilized a qualitative research method to collect data using focus group discussions (FGDs) and in‐depth interviews (IDIs) of high‐school teachers. Participant observations and document reviews were also used to validate the findings from FGDs and IDIs.

### Study site

This study was conducted in Mataram City, Lombok Island, Indonesia. Indonesia is the world’s fourth most populous country (263 991 379 people in 2017)^28^ and people can speak one national language, Bahasa Indonesia (the Indonesian national language). Most Indonesian people are Muslims (87%); other religions are Protestant (7%), Catholicism (3%), Hinduism (2%), Buddhism (1%), and others.^28^ There are also more than 580 other ethnic groups within the nation who speak local languages and exhibit a diverse range of cultures. Mataram City (468 509 people in 2017) is the capital district of West Nusa Tenggara province, located on Lombok Island. Its religions are Islam (96.5%), Hinduism (2.6%), Buddhism (0.3%), Protestant (0.3%), and Catholicism (0.2%)^28^, and it is one of the largest Muslim societies in Indonesia.

### Study participants

Five schools were selected from a total of eight public high schools in Mataram City based on the schools’ academic performance, available facilities, number of students, and economic status of students’ families. In each school, five teachers teaching any of the following subjects were selected to participate in the FGDs – Islamic education, *Pancasila* (state ideology or principles of the state’s philosophy) and civic education, counseling, health education, and homeroom (class) teachers. When there were more than two teachers of the same subject, the one with more experience was invited to participate. IDIs were conducted with principals at each school. Three out of five schools were selected for participant observation, based on their availability.

### FGD and IDI procedure

First, interview guides for FGDs and IDIs were developed. The guide for FGDs was then tested through a pilot FGD. The guide for IDIs was not tested because the main part of these guides shared the same questions.

At the beginning of the FGDs and IDIs, the definition of bullying (Table 1) was confirmed among the participants. In the FGDs and IDIs, the participants were asked how bullying is prevented, how it is recognized, and how they cooperate with other teaching staff. The FGDs and IDIs were conducted in Bahasa Indonesia and the local researchers used the interview guide as a moderator, notetaker, and translator. All FGDs and IDIs were recorded and transcribed with the participants’ consent. Each FGD and IDI lasted for approximately 90 and 60 minutes, respectively, and was terminated when data saturation had been reached – i.e. no additional new data were shared.

**Table 1:** Definitions

Words	Definitions
Bullying	Any unwanted aggressive behavior(s) by another youth or group of youths who are not siblings or current dating partners that involves an observed or perceived power imbalance and is repeated multiple times or is highly likely to be repeated. Bullying may inflict harm or distress on the targeted youth, including physical, psychological, social, or educational harm.^10^, ^29^
Non‐compulsory extracurricular activity	Activities for students that are not included in the curriculum and that take place outside of school hours. Generally, students are not required to participate.
Compulsory extracurricular activity	A type of extracurricular activity for which students’ participation is required. Schools implement these activities regardless of the recommendation from the provincial and/or national government.
Bahasa Indonesia	Indonesian national language, a modified form of Malay.
*Gotong Royong*	Communal work such as cleaning the school.
*Imtaq* (*Iman* and *taqua*)	Lessons on faith and piety.
*Pancasila*	State ideology or principles of the state’s philosophy.
*Toleransi*	Understanding and giving consideration to others.

### Participant observations

The participant observations were conducted through interactions with students, teachers who were not involved in the FGDs, and support staff during classes and other activities on the school grounds. These were carried out at three selected high schools, with one to three visits per school. The observations lasted for 1 to 4 hours at each school. Observations focused mainly on the activities of counseling teachers in their counseling room; the school environment; the general atmosphere of the Islamic education, English, and counseling teacher’s classes; and extracurricular activities including special *imtaq*, daily morning prayers, reading the Qur’an, and flag‐raising. Observational field notes were taken during the observations.

Focus group discussions, IDIs, and participant observations were conducted in February and March 2018.

### Document reviews

Documents deemed relevant to this study were collected from study schools or online to validate the corrected data through FGDs and IDIs between February 2018 and January 2019. Documents written in Bahasa Indonesia were translated into English before the review.

### Data processing and analysis

Data from the FGD and IDI transcripts and audio recordings were transcribed. Because the FGDs and IDIs were conducted in Bahasa Indonesia, the transcripts were translated into English by a native Bahasa Indonesia speaker. Focus group discussion and IDI transcripts and observational field notes were transcribed into MS Word documents and analyzed using thematic analysis. Transcripts were coded in an iterative manner to ensure that all core concepts were included. Initial codes from initial patterns in the data were assigned. Data were then compiled where inferences about the code had been made and themes were generated by multiple researchers. The four‐level social‐ecological model (SEM)^29^ was used as a reference to create themes. Codes and themes were peer reviewed to ensure trustworthiness. Each theme was labeled and defined accordingly.

### Ethical considerations

This study was conducted with approval from the Ethical Committee of University of the Ryukyus (approval number: 381), the Ethical Committee of Mataram University (approval number: 38/UN18.8/ETIK/2015), and the West Nusa Tenggara Provincial Government Regional Development and Research Planning Agency (approval number: 070/ 080/ 02). Written consent was obtained from all participants in the FGDs and IDIs.

## Results

### Sociodemographic characteristics of the respondents

A total of 25 teachers (17 females and eight males) participated in the FGDs. They had 15.6 years of teaching experience on average (range: 2–31 years). Four out of the five IDI respondents were male, and their average teaching experience was 31.2 years (range: 23–41 years).

### Document reviews

The researchers collected and reviewed seven documents: (i) Constitution of the Republic of Indonesia: Article number 28 B of 1945; (ii) the Law of Republic of Indonesia: number 23 of 2017, number 82 of 2015, and number 70 of 2013; (iii) the Ministry of Education and Culture Regulation: number 22 of 2018, Curriculum 2013 and Curriculum 2006; (iv) the Regional Regulation of the Province of Nusa Tenggara Barat: number 4 of 2015; (v) Syllabi: *Pancasila* & Civics Education and Islamic Education; (vi) school rules from four schools; and (vii) school pamphlets from three schools.

### Thematic analysis

The thematic analysis identified 103 codes, 14 categories, and seven themes, namely: (i) Bullying as a problem; (ii) Causes of bullying; (iii) Effects of bullying; (iv) Curricular interventions; (v) Cultural interventions; (vi) Institutional interventions, and (vii) Challenges and recommendations regarding current interventions.

#### Bullying as a problem

Bahasa Indonesia does not have an exact translation of the word “bullying.” It was described as an unpleasant treatment of others and a violation of one’s human rights. Proper handling of bullying cases was considered important and its responsibility lies with schools. If bullying cases left unattended or inadequately addressed, bullying can become a serious problem.

#### Causes of bullying

Physical and psychological disorders among potential victims have been mentioned as triggers for students to initiate bullying.

#### Effects of bullying

Bullying has physical and psychological effects, with the latter being considered to have more impact on the victims. Moreover, the psychological effects may go beyond the victims, even reaching their parents. Bullying may have a complex impact on society. It may go beyond schools, with bullies sometimes ending up as criminals. As for the victims, some could commit suicide. On the other hand, it could be an opportunity to improve the victim’s behaviors, such as those related to personal hygiene.

#### Curricular interventions

*Pancasila* and civics education, and religious education, are compulsory subjects. *Pancasila* and civics education aim to encourage students to be religious and to promote values such as honest behavior, discipline, responsibility, caring, cooperation, peace, courtesy, and responsiveness. Religious education functions to prepare students to become community members who understand and practice religious values and / or acquire expertise in religious studies. Islamic education includes bullying prevention, while *Pancasila* and civics education focuses on character education.

#### Cultural interventions

Cultural interventions include cultural practices, extracurricular activities, and personal contributions of both teachers and principals in bullying‐prevention activities. Bullying is unacceptable in Indonesia and is not in accordance with religious teachings. Different religions exist on Lombok Island and its diversity promotes respect across different ethnic groups. In schools, students practice cooperation, mutual respect, and *toleransi*. *Toleransi* is described as “trying to understand and respect different beliefs and religions.” The schools in this study conduct compulsory extracurricular activities that aim to promote togetherness among students.

#### Institutional interventions

Cases of bullying are best managed within the school when they happen, with the assistance of external stakeholders such as the police and healthcare facilities. The government also engages in advocacy activities. A good physical environment in schools was described as a form of bullying prevention. The schools in this study had similar school rules related to bullying prevention such as punishing bullies. Each school also has its own protocol for dealing with cases of bullying. Overall, bullying prevention was considered a responsibility of teachers. There have been advocacy activities for bullying prevention in the schools, such as posters on safe schools giving helplines.

#### Challenges and recommendations regarding current interventions

Group activities promoting mutual support, togetherness, and understanding among different cultures were believed to help in bullying prevention. However, poor evaluation of school‐level interventions was pointed out as a challenge. The need for additional interventions such as socialization, external linkages, and policies specific to bullying prevention at the national and school levels, was suggested by many teachers. Existing national policies related to bullying prevention should be disseminated to public. It is hoped to establish specific national guidelines for bullying prevention. High school teachers who participated in the study felt that schools can be pioneers in bullying prevention. In addition, school bullying prevention should start in primary school.

The details of the themes are shown in Table 2.

**Table 2:** Seven themes and details

Themes	Reference remarks
**(i) Bullying as a problem** The results revealed that there is no Bahasa Indonesia translation for bullying. Bullying was described as an unpleasant treatment of others and was identified as a violation of one’s human rights. This can be physical or verbal, and can be carried out either individually or by a group. Making racist remarks was regarded as severe bullying. Furthermore, social media could contribute to school bullying (cyberbullying), which is difficult to monitor and control. The respondents identified school bullying as a problem. However, from the teachers’ perspective, bullying can be easily managed. Proper handling of bullying cases was considered important. It was believed that this responsibility should be given to the school and not to the government. If left unattended or inadequately addressed, bullying can become a serious problem.	
**(ii) Causes of bullying** Physical and psychological disorders among potential victims have been mentioned as triggers for students to initiate bullying. A teacher explained how bullying may occur at school and what may happen to the bullied student. [a]	[a] *"In a school setting, there can be a group of students who look down, mock, or vilify others because of their disability. The bullied students become insecure, helpless, inferior, and so on.”* (Islamic education teacher in FGD)
**(iii) Effects of bullying** Many teachers mentioned that bullying has physical and psychological effects, with the latter being considered to have more impact on the victims. Bullied students tend to have lower self‐esteem, feel abused or insecure, and be isolated. Moreover, the psychological effects may go beyond the victims, even reaching their parents. According to teachers, bullied students also tend to have poorer academic achievement and even drop out of school. On the other hand, it was discussed that bullying could be an opportunity to improve the victim’s practices, such as those related to personal hygiene. [b] Teachers stated that, if left unresolved, bullying may have a complex impact on society. Bullying may go beyond schools, with bullies sometimes ending up as criminals. As for the victims, some could commit suicide. [c]	[b] *"In a bullying case that I handled yesterday, a student was bullied because of his body odor. But there was a positive impact too since the student cleaned up after that.”* (Homeroom teacher in FGD) [c] “*From a social standpoint, bullying is not good since we live with other people. It becomes a disease of society if people are being demeaned.”* (*Pancasila* and civics education teacher in FGD)
**(iv) Curricular interventions** Current curricula are competency based, and their structure consists of learning content that should be completed in one level of education for 3 years starting from grades X to XII. There are nine compulsory subjects, including *Pancasila* and civic education, as well as religious education, in accordance with the law. According to the curriculum, *Pancasila* and civics education is provided to students across all grades for two hours per week, while religious education is delivered three hours per week. *Pancasila* and civic education, aims to encourage students to be religious and to promote values such as honest behavior, discipline, responsibility, caring (*gotong royong*, cooperation, *toleransi*, peace), courtesy, and responsiveness. This also promotes a proactive attitude towards being part of the solution to various problems by interacting effectively with the social and natural environment and in positioning oneself as a reflection of the nation to the world. Religious education functions to prepare learners to become community members who understand and practice religious values and / or acquire expertise in religious studies. The respondents also said that Islamic education includes bullying prevention, while *Pancasila* and civic education focuses on character education. Character education is taught by all teachers and the idea of *toleransi* is integrated across lectures according to a national character education policy. Character education is delivered to students through learning across subjects and extracurricular activities, the creation of an educational unit culture, and habituation. The 2013 curriculum also requires students to participate in scouting activities (being Boy or Girl Scouts). One of the teachers referred to character education in the curriculum. [d]	[d] “*In character education, we are expected to teach basic competencies such as values of independence, responsibility, cooperation, and discipline. These values must be incorporated into the syllabus.”* (Homeroom teacher in FGD)
**(v) Cultural interventions** Cultural interventions include cultural practices, extracurricular activities, and personal contributions of both teachers and principals in bullying prevention activities. Teachers said that bullying is unacceptable in Indonesia. It is also not in accordance with religious teachings. There are different religions on Lombok Island. This diversity promotes respect across different ethnic groups. In schools, students practice cooperation, mutual respect, and *toleransi*. *Toleransi*, as described by one of the respondents, is “trying to understand and respect different beliefs and religions. We should not disturb others.” [e] In addition to the formal curriculum, a number of non‐religious‐oriented schools implement religious activities, such as collective prayers and religious teaching in extracurricular activities. A regional regulation states that the education units must offer extracurricular activities and that activities are directed toward developing local cultural arts, expertise, and character education, and increasing nationalism. The schools involved in this study also conduct compulsory extracurricular activities that aim to promote togetherness among students. These include: (i) general *imtaq* (lessons on faith and piety) for all students; (ii) special *imtaq* specific to each religious group; (iii) flag‐raising ceremony; (iv) *Gotong Royong* (communal work such as cleaning the school); (v) daily morning prayers; and (vi) reading the Qur’an in class. These teach students how to improve their character, particularly in terms of respecting others, and how they can contribute both to their school and society. All these activities are considered to promote togetherness among students. Furthermore, these are all compulsory extracurricular activities. A regional regulation states every educational unit must apply religious practices such as *Imtaq* activities, which are held every Friday before the lesson begins, and the reading and writing of the Qur'an for graduates of Islamic primary and secondary education units and adapted non‐Muslim students. *Gotong royong* and flag‐raising ceremonies should be arranged at schools. The Ministry of Education and Culture regulation has guidelines for flag‐raising ceremonies. The ceremony aims to: (i) strengthen the unity of the nation and the unitary state of the Republic of Indonesia; (ii) help students to become orderly and disciplined; (iii) improve leadership skills; (iv) instill solidarity and cooperation; (v) foster a sense of responsibility; and (vi) strengthen the spirit of nationalism and love for country. [f] Non‐compulsory extracurricular activities were also believed to be effective in bullying prevention among schools. For example, scouting activities may improve student behavior, specifically by molding students to become more disciplined. Flag‐raising ceremonies nurture nationalism and regionalism. Other activities include the Red Cross, drum band, theater club, journalism club, English debate, cultural clubs (i.e., Dutch, Japanese), sports (football, volleyball, basketball, Karate) clubs, religious clubs (Muslims, Hindus), etc. [g] Teachers shared their contribution to bullying prevention, such as encouraging students to practice *toleransi* and promoting togetherness among them. They also guide students to behave based on ethics and norms. Teachers emphasize the importance of discipline, motivate students to be better, and ask them to be vocal about their feelings or opinions. They also help improve students’ character and self‐esteem by molding them to be independent. Teachers believe that it is important for their students to be able to solve problems themselves. Teachers also demonstrate the values that they advocate among their students. They practice good character, listen openly to students, show their affection for the students, and build mutual respect and togetherness. During morning greetings, teachers welcome their students and greet them with a smile at the school gate. These efforts make students feel appreciated, as they are acknowledged by their teachers. Moreover, teachers monitor student behavior to see if there is a need for improvement. One of the teachers described how they impact their students. [h] Furthermore, teachers provided additional support to students who are more likely to be bullied, such as those with special needs. They talk to the concerned parents to learn more about them, particularly how the students are doing at home. This allows the teachers to come up with holistic interventions that cover both school and home settings.	[e] *“Through class activities, they learn how to appreciate diversity. They see diversity as an opportunity to live in harmony. They can live together with respect.”* (Counseling teacher in FGD) [f]“*School rules state that students are not allowed to take any harmful actions, including bullying. Aside from that, we also convey messages at general imtaq. In this activity, we highlight that all of us are brothers, so no one should insult or mock others.”* (Principal in IDI) [g] *“I'm the adviser of the student council. Aside from the council, there are many extracurricular activities in this school. We hope that these activities will guide students to the positive direction. If they are passionate in these activities, they don't think of negative things.”* (*Pancasila* and civics education teacher in FGD*)* [h] “*In fact, it doesn't matter whether the teachers know what disciplinary actions are stated in the school rules or not. We take on the responsibility of disciplining students because that's our role as teachers. We are educators who will be imitated or copied by students.”* (Homeroom teacher in FGD)
**(vi) Institutional interventions** Cases of bullying are best managed within the school when these happen, with the assistance of external stakeholders such as the police and healthcare facilities. The government also engages in advocacy activities. A good physical environment in schools was described as a form of bullying prevention. [i] The schools involved in this study had similar school rules. The rules related to bullying prevention were mentioned. An example is punishing bullies. If bullying occurred repeatedly, the students would be expelled. Each school also has its own protocol for dealing with cases of bullying. Usually, the teacher who witnessed the bullying incident will manage the case. If the situation is not resolved, the next step is to report the case to the vice‐principal handling student affairs and then the principal. Parents will become involved when the case becomes unmanageable or if it involves cyberbullying. In some schools, the responding teacher asks for help from his / her co‐teachers before going to the vice‐principal. Overall, bullying prevention was considered a responsibility of teachers. When it comes to monitoring, teachers watch out for possible signs of bullying such as behaviors and student interactions in classes and other school activities. Some students also seek help directly from their teachers if they or their friends are being bullied. The schools work with external parties such as hospitals and police units. They invite them to serve as resource persons for student activities, particularly in dealing with cases of bullying. The police are also involved if there is a case of violence. The police also patrol schools within their assigned areas. One of the teachers shared an experience with the police being resource persons. [j] There were advocacy activities for bullying prevention in the schools. Some of the schools posted a sign stating “Safe school,” which mentions all types of violence, including bullying. The sign also included SOS contacts whom students may approach for help. According to the teachers, calls to prevent bullying were featured in pamphlets, social media, and television advertisements. [k]	[i]“*A beautiful environment is the first step to change students who have unclean spirits possibly caused by living in a dirty house. So, if they stay in a clean school, eventually the nature of their aggressiveness slightly diminishes.”* (Counselling teacher in FGD) [j] *“Resource persons from the police department discuss how to create a safe community in schools or how to avoid getting involved in crimes.”* (*Pancasila* and civics education teacher in FGD) [k] “*I have not seen or heard any regulations on bullying prevention from the government but there are anti‐bullying advertisements like slogans and stickers on social media or television. It seems like the government has started to address the problem.”* (Counseling teacher in FGD)
**(vii) Challenges and recommendations regarding current interventions** Group activities promoting mutual support, togetherness, and understanding among different cultures were believed to help in bullying prevention. Generally, bullying prevention activities were perceived to be successful. One of the teachers explained the effectiveness of their interventions. [l] The impact of extracurricular activities on preventing bullying was discussed among the teachers. Nevertheless, not all students join extracurricular activities. Poor evaluation of school‐level interventions was pointed out as a challenge in the current interventions. The need for further consideration of bullying prevention was suggested by many teachers. Specifically, socialization, external linkages, and policies specific to bullying prevention at the national and school levels were some of the suggestions shared. However, one of the teachers mentioned that existing national policies related to bullying prevention should be widely disseminated to the public. One of the teachers expressed hope for the establishment of specific national guidelines for bullying prevention. [m] Teachers felt that schools can be pioneers in bullying prevention. In addition, school bullying prevention should start in primary school, as teachers were aware that the impacts of bullying can increase over time. They also acknowledge the importance of them being able to understand their students.	[l] *“(Intervention is) very successful because it seems like bullying rarely happens. There is a sense of family who loves each other, respects each other, helps each other, mutual* toleransi*, and mutual openness. InsyaAllah (God‐willing). We must encourage children to be open.”* (Counselling teacher in FGD) [m] “*Each school has different ways of handling bullying cases at the moment. I think it would be nice if there was specific technical guidance or specific guidelines from the central government so that all schools in Indonesia will be able to make more integrated efforts in bullying prevention.”* (*Pancasila* and civics education teacher in FGD)

## Discussion

From the study findings, two major interventions on bullying prevention in public high schools in Lombok Island were identified – curricular and extracurricular interventions. Curricular education is what students learn in the classroom. Extracurricular activities are opportunities to practice what they have learned. Both are based on their culture. Aside from these interventions, schools provide an enabling environment for bullying prevention.

The connection between education, culture, and religion is very strong in these Indonesian curriculum documents.^30^ Our study suggests that these interactions effectively influence the prevention of bullying. *Pancasila* and civics education, as well as religious education, are included in the school curriculum as a subject, while character education is integrated into the whole learning process.^31^ These lessons include bullying prevention. This demonstrates ownership by both the government and schools, which was also identified as a factor in successful implementation of school health.^32^ The Education Law of the Republic of Indonesia states that the national education is based on *Pancasila. Pancasila* and consists of five principles: (i) belief in one God; (ii) just and civilized humanity, including tolerance of all people; (iii) unity of Indonesia; (iv) democracy led by the wisdom of deliberation among representatives of the people; and (v) social justice for all. *Pancasila* and civics education covers topics such as principles and practices of conflict resolution, tolerating differences, respect for individual rights, and participatory instruction.^33^ The present study emphasized the importance of character education.^34^ Character education is highlighted in the Indonesian government’s Long‐Term Development Plan for 2005–2025.^35^ The study findings indicate that improvement in student character prevents school bullying. Previous research also concluded that character education is effective in bullying prevention.^36^

Islamic education and other types of religious education are unique in Indonesia. Religious education is not specialized and is incorporated into general education in combination with moral education. Other types of religious education are officially approved. It is also thought that these curricula are effective in preventing bullying. In many countries, degrees from Islamic education institutions are not recognized by larger education systems or general education systems, and Islamic education is usually limited in general schools.^37^ On the other hand, in Indonesia, Islamic education in government schools is recognized as an equivalent to religious education.^38^ As with other religious education subjects, Islamic education is required to be taken by students.^39^

Our study findings show that extracurricular activities, including cultural events, can also prevent bullying. All interventions are influenced by their culture, especially the practice of *Toleransi* and religious values. Most of the teachers commonly practice cultural interventions and believe that these are effective in bullying prevention. The SEM can be used as a framework for the prevention of violence, including school bullying.^29^ To prevent bullying, it is necessary to act across several different levels (individual, relationship, community, and society) at the same time.^40^ The SEM helps identify the prevention strategies that can be used at each level to address these factors. This approach is more likely to sustain prevention efforts over time than a single intervention.^41^ To obtain a better understanding of the study findings, we used SEM to describe the relationship between each type of intervention. At the individual level, all the interventions are applicable, specifically, promoting students’ attitudes, beliefs, and behaviors. At the relationship level, cultural interventions are applicable, molding students to be independent so that they can solve their problems by themselves, promoting healthy relationships between peers and teachers, and supporting students in making friends and cooperating with their parents. Institutional interventions at the community level include maintaining a good physical environment in schools and having school rules related to bullying prevention. At the societal level, societal and cultural norms are taught in the curriculum and teachers guide students in behaving in accordance with ethics and norms. Some of the key elements, especially at the community and societal levels, were not identified because this study focused on school settings.

Lombok Island is culturally diverse. Indonesia has more than 580 ethnic groups who speak local languages and exhibit a diverse range of cultures. The number of members of each group ranges from several hundred to millions.^42^ There are two main ethnicities on Lombok Island: the Sasak and the Balinese. A previous study revealed that informal cultural ties play a significant role in maintaining the integration, and social unity between the two communities in Mataram City, Lombok Island.^43^

Extracurricular activities can be non‐compulsory or compulsory. The former activities are open to all students, who are encouraged to join, although participation is optional. The latter activities are mandatory for all students. Participating in these activities promotes interaction and mutual understanding among students. Previous studies have revealed the effectiveness of cultural interventions. First, there are several reports (from multiple settings, in various countries, and in areas of different faiths) on using religion as a public health intervention to improve health outcomes.^44^ Second, previous studies suggested that, to be optimally effective, school‐based prevention programs should be sensitive to participants’ cultural, ethnic, and socio‐economic backgrounds.^45^, ^46^, ^47^ Finally, previous studies in Indonesia suggested that effective school‐based programs consider cultural practices, values, and traditions.^48^, ^49^

Previous studies also support the impact of religious education and religious practices.^50^, ^51^ Religious practices and religious involvement have been proven to help individuals deal with stressful situations, anxiety, and isolation, among others.^52^ Teaching religion in secular schools can provide a student with a deeper understanding of different cultures around the world and allow the student to acquire values that they can integrate into their own life.^53^ Religious education strengthens the formation of moral consciousness through the internalization of religious morality.^54^

The Health Promoting Schools (HPS) initiative has been recognized as playing an important role in promoting positive development and healthy behaviors such as physical activity, balanced nutrition, the prevention of tobacco use, and the prevention of bullying.^55^ This indicates that the interventions found in this study play an important role beyond bullying prevention in a school setting. The previous study suggested that creating a good physical and social environment contributes to the reduction of violence or key risk factors for violence and bullying.^56^, ^57^ Our study has also found an approach that leads to the creation of a good social environment through curricular and extracurricular activities.

It is necessary to conduct a similar study in primary education to confirm the findings obtained in this study. In general, the age group that bullying is most prevalent in is at primary school. However, we decided to avoid observational studies among young pupils due to ethical considerations. It was found that religious education and moral education are effective in preventing bullying, and they are implemented systematically from the primary level in Indonesia. Information was obtained in this study through interviews with teachers and participant observations in schools. Thus, even without delving into direct observation about bullying among pupils, which is likely to raise research ethics issues, the effects of religious and moral education could be described.

One of the limitations of thematic analysis is that it explores events in depth, which limits the generalizability of the research results. The study area was selected to be distinctive in terms of religious background in Indonesia. Residents of Mataram city are predominantly Muslim, but the population is culturally diverse with residents practicing other religions such as Hinduism. The city is also ethnically diverse.

### Conclusions

*Pancasila* and civics education and religious education teach values related to bullying prevention. Character education that includes bullying prevention is integrated into the curriculum. The study findings strongly support the importance of promoting a school‐based bullying prevention program, especially in the curriculum. Cultural practices and extracurricular activities allow students to demonstrate the values that they have learned. Schools also implement policies and maintain a good physical environment so that students do not bully their peers.

Current interventions in Indonesia should be continued and must be modified to respond to societal changes both at the country and international levels. Countries should consider promoting a school‐based bullying prevention program through revision of their academic curriculum. Moreover, it is important to integrate religious education and character education while creating HPS, as these types of education contribute to overall positive health outcomes.

## Disclosure

This study was supported by the Grant for the National Center for Global Health and Medicine (30‐3, 30‐4), Gender Equality Promotion Office, University of the Ryukyus and the University of the Ryukyus Foundation. The sponsors did not play any role in this study. The authors declare no conflict of interest.

## Author contribution

T.N. and J.K. designed the study. J.K. conceived of the idea. T.N., E.A., L.N., and W.S.A. collected the data. T.N. and P.M.R.H. analyzed the data. P.M.R.H., D.N., H.K., and J.K. provided technical advice. T.N., P.M.R.H., and R.T. drafted the manuscript. J.K. critically reviewed the manuscript. All authors read and approved the final manuscript.
